# Nematode community responses to range‐expanding and native plant communities in original and new range soils

**DOI:** 10.1002/ece3.4505

**Published:** 2018-10-02

**Authors:** Rutger A. Wilschut, Olga Kostenko, Kadri Koorem, Wim H. van der Putten

**Affiliations:** ^1^ Department of Terrestrial Ecology Netherlands Institute of Ecology (NIOO‐KNAW) Wageningen The Netherlands; ^2^ Laboratory of Nematology Wageningen University Wageningen The Netherlands; ^3^ Department of Botany Institute of Ecology and Earth Sciences University of Tartu Tartu Estonia

**Keywords:** novel interactions, plant–nematode interactions, plant–parasitic nematodes, range‐expanding plant species, root‐feeding nematodes

## Abstract

Many plant species expand their range to higher latitudes in response to climate change. However, it is poorly understood how biotic interactions in the new range differ from interactions in the original range. Here, in a mesocosm experiment, we analyze nematode community responses in original and new range soils to plant communities with either (a) species native in both the original and new range, (b) range‐expanding species related to these natives (related range expanders), or (c) range expanders without native congeneric species in the new range (unrelated range expanders). We hypothesized that nematode community shifts between ranges are strongest for unrelated range expanders and minimal for plant species that are native in both ranges. As a part of these community shifts, we hypothesized that range expanders, but not natives, would accumulate fewer root‐feeding nematodes in their new range compared to their original range. Analyses of responses of nematodes from both original and new ranges and comparison between range expanders with and without close relatives have not been made before. Our study reveals that none of the plant communities experienced evident nematode community shifts between the original and new range. However, in soils from the new range, root‐feeding nematode communities of natives and related range expanders were more similar than in soils from the original range, whereas the nematode community of unrelated range expanders was distinct from the communities of natives and related range expanders in soils from both ranges. The abundances of root‐feeding nematodes were comparable between the original and new range for all plant communities. Unexpectedly, unrelated range expanders overall accumulated most root‐feeding nematodes, whereas related range expanders accumulated fewest. We conclude that nematode communities associated with native and range‐expanding plant species differ between the original and the new range, but that range‐expanding plant species do not accumulate fewer root‐feeding nematodes in their new than in their original range.

## 1 INTRODUCTION

Worldwide, many native plant communities are invaded by exotic species that have been introduced intentionally or unintentionally by humans (van Kleunen et al., 2015). In addition to exotic species that originate from other continents, current climate change enables intracontinental range expansion of plant and animal species to higher latitudes and altitudes (Parmesan, 2006; Walther et al., 2002). While such range expanders have become increasingly common (Tamis, Zelfde, Meijden, & Haes, 2005), little is known about their influence on native above‐ and belowground plant‐associated biota in their novel habitat (Van Nuland, Bailey, & Schweitzer, 2017). The limited co‐evolutionary history may result in naïve responses of either plants or associated biota (Pearse, Harris, Karban, & Sih, 2013; Verhoeven, Biere, Harvey, & van der Putten, 2009), which makes outcomes of such novel interactions difficult to predict.

The success of introduced exotic plant species has often been related to their possession of traits that are not present in the invaded native community. Next to novel traits such as fire resistance (D'Antonio & Vitousek, 1992) and nitrogen fixation (Stock, Wienand, & Baker, 1995), non‐native plant species may also benefit from the production of metabolites that are not produced by native plant species (Cappuccino & Arnason, 2006). In the new range, such “novel weapons” may suppress the growth of neighboring plant species (Callaway & Aschehoug, 2000), mutualists of native species (Callaway et al., 2008; Stinson et al., 2006), and natural enemies (Macel, de Vos, Jansen, van der Putten, & van Dam, 2014; Schaffner et al., 2011). Because plant traits such as root chemistry are often phylogenetically conserved (Agrawal et al., 2009; Gilbert & Parker, 2016; Pearse & Hipp, 2009), exotic species that are phylogenetically closely related to native flora may host more natural enemies in the invaded range than distantly related range expanders (Gilbert & Parker, 2016). These, so‐called spillover effects of local enemies (Malmstrom, McCullough, Johnson, Newton, & Borer, 2005) are considered as one of the possible explanations why phylogenetically distinct exotic species can become more abundant than exotic species that are strongly related to native species (Strauss, Webb, & Salamin, 2006).

Some intracontinental range expanders are closely related to plant species in the native plant community, but are nonetheless found to be more successful in suppressing generalist insects, fungal pathogens, and root‐feeding nematodes than their related native species (Engelkes et al., 2008; Morriën, Duyts, & Van der Putten, 2012; Morriën & van der Putten, 2013; Wilschut, Silva, Garbeva, & van der Putten, 2017). Range expanders that are phylogenetically more distinct from native flora can be expected to have even stronger suppressive effects on these native natural enemies, but such evidence is lacking so far. Moreover, it is still largely unknown whether the interactions between range‐expanding plant species and their natural enemies differ between their original and new range as only a couple of studies (Dostálek, Münzbergová, Kladivová, & Macel, 2015; van Grunsven, van der Putten, Bezemer, Berendse, & Veenendaal, 2010; Macel et al., 2017) have addressed these questions experimentally.

The aim of the present study was to examine plant–nematode interactions of natives, range expanders related to these natives (hereafter, related range expanders), and range expanders without native species from the same genus in their new range (hereafter, unrelated range expanders), in soils from the new and original range. We focus on belowground plant–nematode interactions, as nematodes have important roles in the soil food web (Ferris, Bongers, & De Goede, 2001) and can affect spatiotemporal dynamics in natural vegetation (Brinkman, Duyts, Karssen, Van der Stoel, & Van der Putten, 2015; De Deyn et al., 2003). We established mesocosms with soil from either the original or the new range, in which we grew communities of each of the three groups of plant species. We recorded the abundance of root‐feeding nematodes, as well as bacterivores, fungivores, omnivores, and predators in the root zones of all plant communities growing in soils from the original and the new range.

We tested the hypotheses that (a) range expanders, but not natives, associate with different nematode communities in the original compared to the new range, mostly by accumulating fewer root‐feeding nematodes in soil from their new range; (b) these shifts in nematode communities will be stronger for unrelated than for related range expanders; and (c) we expect that numbers of bacterivorous, fungivorous, omnivorous, and predatory nematodes vary less between the plant communities than root‐feeding nematodes, as they are only indirectly interacting with the plants (De Deyn, Raaijmakers, Van Ruijven, Berendse, & Van Der Putten, 2004; Scherber et al., 2010).

## 2 METHODS

We tested our hypotheses using three types of plant communities consisting either of (a) four plant species that are native in both southeastern Europe, where the range expanders originate from, and northwestern Europe, where range expanders have expanded to; (b) four plant species belonging to the same genera as the natives and that have expanded their range from southeastern Europe to northwestern Europe; or (c) four plant species that have expanded their range from southeastern Europe to northwestern Europe and have no native species in the same genus in the new range. In a greenhouse experiment, we grew all three plant communities in mesocosms with a sterilized background soil, inoculated with individual replicates of soil from either the original or the new range (see below). After a growth period of 14 weeks, we extracted the nematode communities from the soil of each mesocosm for counting and identification.

### 2.1 Plant species and seed collection

All plant species occur in central Netherlands in riparian habitats of the three rivers that are branches of the Rhine. The majority of these plant species can be found in the same nature reserves (Dutch nature observation website: https://www.waarneming.nl). The native plant species were *Centaurea jacea* L. (Asteraceae), *Tragopogon pratensis* L. (Asteraceae), *Geranium molle* L. (Geraniaceae), and *Rorippa sylvestris* (L.) Besser (Brassicaceae). As related range expanders, we used *Centaurea stoebe* L.*, Tragopogon dubius* Scop.*, Geranium pyrenaicum* Burm. f., and *Rorippa austriaca* Crantz. The four unrelated range expanders were *Dittrichia graveolens* (L.) Greuter (Asteraceae)*, Lactuca serriola* L. (Asteraceae), *Rapistrum rugosum* (L.) All. (Brassicaceae), and *Bunias orientalis* L. (Brassicaceae). *Centaurea stoebe, T. dubius, R. austriaca, D. graveolens,* and *R. rugosum* colonized the Netherlands from the 20th or early 21st century onwards, while *G. pyrenaicum, L. serriola,* and *B. orientalis* already occurred in suitable habitats of the Netherlands before the 20th century, but strongly expanded their range during recent decades (NDFF 2017). Seeds of all 12 plant species originated from single, wild populations growing in the Netherlands. For *G. pyrenaicum*,* T. dubius* and *T. pratensis ssp pratensis* seeds were supplied by Cruydthoeck, a company that grows plants from field‐collected seeds in the Netherlands for seed production. For all other plant species, we collected seeds directly from plants growing in natural areas, mainly in riverine systems in eastern Netherlands.

### 2.2 Soil collection

We collected soil from areas in Slovenia and Austria where all plant species occur naturally and from the riverine system in the Netherlands where all the range‐expanding plant species have become established. In all three countries, we selected three riverine areas of approximately 30 ha each for soil collection. The soils in all these riverine areas are comparable as they all are of alpine origin. In each area, soils were collected from three sublocations separated by a distance of minimally 300 m. First, we removed the upper 3 cm soil layer and then collected the soil between 3 and 15 cm depth, where most living roots occur. Thereafter, the soil was sieved using a 4 mm mesh and gently homogenized, while keeping sublocations separate. Half the soil from each sublocation was stored at 4–8°C, while the other half was sterilized by gamma irradiation (>25 KGray) at Steris AST (Ede, The Netherlands). To compare the effects of soil biota under the same abiotic conditions, we used a common sterilized background soil that was a mixture of riverine sandy clay soils additionally collected from all sublocations in the Netherlands. Background soil was sieved, homogenized, and then gamma‐sterilized as indicated above.

### 2.3 Experimental setup

We first created nine soil replicates for both the original and the new range. To obtain soil replicates with communities of soil organisms that represented the new and original range in a general and not location‐specific way, each of these nine replicate soils consisted of sterilized background soil to which live soil from two sublocations, originating from two different main areas in either the original or the new range, was inoculated (see Koorem et al., 2017). This approach resulted in nine soil mixes that were nonidentical, yet partly overlapping in donor soils, and avoided the risk of idiosyncratic differences among individual soil samples. All soils were collected from sites where at least several of the plant species that were used in the experiment occurred. However, to avoid that soil mixes were dominated by soil biota associated with one of the focal plant species, we did not collect soil directly beneath these plant species. The soil mixes representing soils from the original range were a combination of soil from one of the nine Slovenian and one of the nine Austrian sublocations (see Supporting Information Appendix S1). For the new range, nine soil mixes were created by combining soils from two different locations in the riverine system in the Netherlands (see Supporting Information Appendix S1), so that each sublocation was used in two different soil mixes. Each mesocosm (7 L, diameter 26 cm, height 20 cm) in the experiment was filled with 1.5 kg of gravel (4–8 mm particles) at the bottom on top of which 4.2 kg of soil was added, consisting of 80% sterilized background soil and 10% live soil inoculum from the two sublocations. To avoid potential abiotic differences between soils from the original and the new ranges, we added 10% of sterilized inoculum soil from the complementary range, so that in all cases every mesocosm had 10% of (sterilized or unsterilized, respectively) soil from the original and 10% from the new range.

Per range, each of the nine soil mixes was divided over three different mesocosms, resulting in 54 mesocosms (nine soil mixes × three plant communities × two soil origins) in total. Each mesocosm was planted with two seedlings of each of the four plant species of the same plant type in the Netherlands, so that on each soil mix all three plant communities were grown. Seedlings were planted in a circle in a fixed order at approximately 4 cm of each other, in such a way that conspecific seedlings were not close neighbors. Mesocosms were placed in a climate‐controlled greenhouse of 16 hr 21° (day) and 8 hr 16° (night) and were watered three times per week in order to keep soil moisture at 60% water‐holding capacity. Every week, the mesocosms were moved to a different position in the greenhouse in order to avoid variation caused by differences in greenhouse conditions. We did not add any nutrients to the mesocosms throughout the growth period. After 12 weeks of plant growth, two *Mamestra brassicae* L. (Lepidoptera: Noctuidae) caterpillars were introduced to pots with the soil replicates 1–5 of both new and original range soils (Supporting Information Appendix S1) in order to test their response to the different plant communities (see Koorem et al., 2017). We did not aim to test the effects of aboveground herbivory on nematode community composition. The herbivory treatment was assigned to soil mixes 1–5 (Supporting Information Appendix S1), which due to their origin likely more closely resemble each other than they resemble soil mixes 6–9. For example, original range soil mixes 1–3 share soil from the same sublocation in Austria, whereas mixes 7–9 all share soil from a different sublocation. Because of this nonrandom assignment of the herbivory treatment, it is impossible to disentangle herbivory effects from soil mix effects in the presented study.

### 2.4 Harvest

After 14 weeks of growth, shoots of all individual plants were clipped, dried at 70°C, and weighed. As it was not possible to disentangle the roots of each individual plant, roots of all plants were washed from the soil collectively and dried at 70°C to constant weight. We used 50 g of soil (wet weight) from each pot for nematode extraction, morphological identification, and counting to feeding type. Additionally, soil samples were taken for determining soil moisture content, so that the number of nematodes could be expressed per dry weight of soil. Nematodes were extracted from soil using an Oostenbrink elutriator (Oostenbrink 1960). After extraction, we concentrated the nematode suspensions to 2 ml, after which 4 ml hot (90°C) and 4 ml cold (20°C) formaldehyde was added to fixate the nematodes before identification and counting.

### 2.5 Nematode identification

Morphological identification and counting of nematodes were done using an inverse‐light microscope at 200× magnification. Per sample, all nematodes were classified to one of the five feeding types (predators, root feeders, fungivores, omnivores, or bacterivores) according to Yeates, Bongers, Degoede, Freckman, and Georgieva (1993) and counted. Root‐feeding nematodes were further identified to either family or genus level using Bongers (1988). Root‐feeding nematode genera identified were *Meloidogyne* (Heteroderidae), *Paratylenchus* (Tylenchulidae), *Pratylenchus* (Pratylenchidae), and *Psilenchus* (Psilenchidae), and root‐feeding nematode families identified were Hoplolaimidae*,* Tylenchidae*,* Anguinidae*,* Dolichodoridae*,* Criconomatidae*,* Hemicycliophoridae, and Heteroderidae.

### 2.6 Statistical analyses

Prior to statistical analyses, soil moisture percentages were used to calculate nematode numbers per 100 g dry soil. We also calculated the density of root‐feeding nematode taxa per gram root, as an indication of the root‐feeding nematode density on plant roots. For this, we calculated total number of nematodes of each taxon per mesocosm and divided those numbers by total root dry weight in that mesocosm (Supporting Information Appendix S2; also presented in Koorem et al., 2017).

#### 2.6.1 Multivariate analyses

First, we performed a principal component analysis (PCA in Canoco 5; Šmilauer and Lepš, 2014) comparing nematode community composition based on the abundances of the five nematode feeding types. Second, in another PCA analysis, we compared community composition of only the root‐feeding nematode community, as root‐feeding nematodes were expected to show the strongest responses to plant status in the Netherlands. Nematode taxa with fewer than three occurrences in the data set were excluded from the analyses to avoid strong effects caused by rare taxa. We used the factors “plant community” and “soil origin” to independently classify the mesocosms. In both PCAs, we included soil mix as a covariate in order to account for variation between the nine soil mixes. To test the effects of plant community, soil origin, and their interaction on the nematode community composition, we used individual redundancy analyses (RDA) in Canoco 5 for both the main effects and the interaction effect. The significance of the RDA models is based on 999 Monte Carlo permutations, which were restricted to incorporate the effect of soil mix.

#### 2.6.2 Univariate analyses

All univariate analyses were performed in R version 3.1.0 (R Core Development Team 2012). We selected four nematode feeding types and four root‐feeding nematode genera/families that—based on the PCA—contributed most to the separation of the treatments. Densities of predators were so low (average 1.27 per sample) that they were not modeled. We used generalized linear models with a negative binomial error distribution (Hilbe, 2014) to model densities of the nematode feeding types in soil (N/100 g soil). Similar models were used to model densities of the selected root‐feeding nematode genera and families in soil (N/100 g soil) and per g root (N/g root). Generalized linear models included the fixed factors, soil mix (nested in soil origin), plant community, soil origin, and the soil origin*plant community interaction. Post hoc Wald tests were performed using the phia package (De Rosario‐Martinez, 2013) to individually test differences between plant communities.

## 3 RESULTS

### 3.1 Nematode feeding type community composition

**Figure 1:**
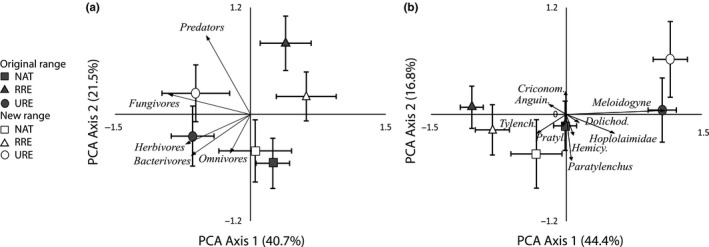
Ordination diagrams of principal component analyses (PCA) showing the centroids of nematode community composition based on nematode feeding types (left) and the community of root‐feeding nematodes (right). Centroids represent nematode communities in mesocosms inoculated with soils from the original range (filled signs) or new range (open signs), grown with either natives (NAT; squares), related range expanders (RRE; triangles), or unrelated range expanders (URE; circles). Arrows represent the relation between nematode feeding types (a) or between root‐feeding nematode taxa (b) and the variation in nematode community along the PCA axes. Horizontal and vertical error bars represent standard errors along the first and second PCA axes. Percentages of total explained variation by the PCA axes are given in the parentheses

The nematode community composition based on feeding types was significantly affected by the interaction between plant community and soil origin (RDA: total variation explained: 22.1%; pseudo‐*F* = 2.7, *p* = 0.003). In particular, the nematode communities accumulated by related range expanders differed between soils from the original range and soils from the new range, while nematode communities accumulated by natives and unrelated range expanders did not differ between original and new range soils (Figure 1a).

### 3.2 Root‐feeding nematode community composition

The community composition of root‐feeding nematodes was affected by the interaction between plant community and soil origin (RDA: total variation explained: 21.4%; pseudo‐*F* = 2.6, *p* = 0.001, Figure 1b). In particular, all three plant communities had differently composed root‐feeding nematode communities. However, in the original range, nematode communities of native and related range expanders were more strongly separated than in the new range. In contrast, the nematode community of the unrelated range expanders was more separated from the other nematode communities in the new range compared to the old range. The root‐feeding nematode groups that contributed most strongly to the separation between the treatments were *Meloidogyne*,* Paratylenchus, Hoplolaimidae,* and *Tylenchidae*.

### 3.3 Abundances of the nematode feeding types

**Figure 2:**
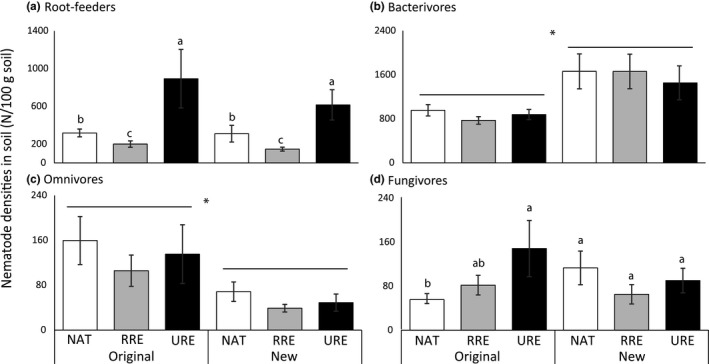
Densities of root‐feeding (a), bacterivorous (b), omnivorous (c), fungivorous (d), and nematodes in soil (N/100 g dry soil) in mesocosms with native plant species (NAT; white), related range expanders (RRE; light grey), and unrelated range expanders (URE; dark grey) in soils from the original range (south) and the new range (north) of the range expanders. Bars represent averages ± standard errors. Horizontal bars and asterisks indicate significant differences between soil origins and different letters indicate significant (*p* < 0.05) differences between plant communities within ranges based on negative binomial GLM and post hoc Wald tests

Differences in densities of root‐feeding nematodes (N/100 g soil) were solely explained by plant community type (explained deviance: 44.55, *p*(*χ* ^2^, *df* = 2) < 0.0001; Figure 2a). Overall, unrelated range expanders accumulated more root‐feeding nematodes (N/100 g soil) than natives (*χ* ^2^ = 14.74, *p* < 0.001) and related range expanders (*χ* ^2^ = 43.63, *p* < 0.0001), whereas natives accumulated more root‐feeding nematodes than their related range expanders (*χ* ^2^ = 7.69, *p* < 0.01). Numbers of bacterivorous and omnivorous nematodes (N/100 g soil) differed between original and new range soil: Bacterivorous nematodes were most abundant in soil from the new range (explained deviance: 22.32, *p*(*χ* ^2^, *df* = 1) < 0.0001; Figure 2b), whereas omnivorous nematodes (N/100 g soil) were most abundant in soils from the original range (explained deviance: 26.81, *p*(*χ* ^2^, *df* = 1) < 0.0001; Figure 2c). The numbers of fungivores (N/100 g soil) depended on the interaction between soil origin and plant community type (explained deviance: 6.11, *p*(*χ* ^2^, *df* = 2) < 0.05). In soils from the original range, fungivore densities (N/100 g soil) were higher in mesocosms with unrelated range expanders than with native plant species (*χ* ^2 ^= 7.13, *p* < 0.01; Figure 2d), whereas there were no differences between plant community types in soils from the new range.

### 3.4 Abundances of root‐feeding nematodes

**Figure 3:**
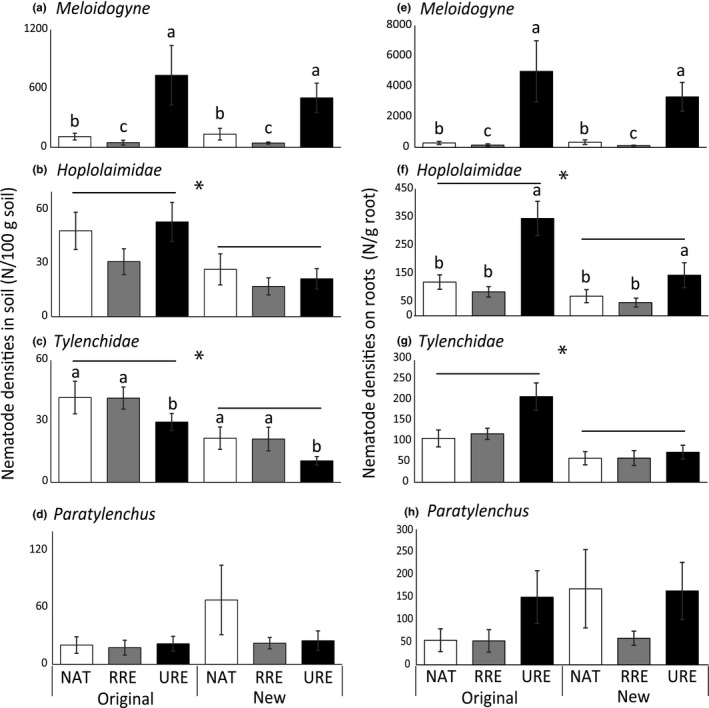
Plant community effects on densities in soil (N/100 g dry soil; left) and on roots (N/g root; right) of root‐feeding nematode groups *Meloidogyne* (a, e), *Hoplolaimidae* (b, f), *Tylenchidae* (c, g), and *Paratylenchus* (d, h) in soils from the original range and new range. Different bars represent the communities of native plants (NAT; white), related range expanders (RRE; light gray), and unrelated range expanders (URE; dark gray). Bars represent averages ± standard errors. Horizontal bars and asterisks represent significant (*p* < 0.05) differences between soil origins, and different letters indicate significant (*p* < 0.05) differences between plant communities within the ranges based on negative binomial GLM and post hoc Wald tests

Responses of all root‐feeding nematodes to soil origin and plant community composition depended on genus/family and whether nematode numbers were analyzed per 100 g soil or per g root (Figure 3). *Meloidogyne* was the most abundant root feeder, as 44% of the root‐feeding nematodes in the mesocosms with natives, and 30% with related and 82% with unrelated range expanders belonged to this genus. *Meloidogyne* densities were strongly affected by plant community type (N/100 g soil: explained deviance: 55.15; *p*(*χ* ^2^, *df* = 2) < 0.0001; N/g root: explained deviance: 99.82; *p*(*χ* ^2^, *df* = 2) < 0.0001; Figure 3a,e). Densities of *Meloidogyne* in soil, as well as *Meloidogyne* densities on roots, were higher in mesocosms with unrelated range expanders than with natives (N/100 g soil: *χ* ^2^ = 21.35, *p* < 0.0001; N/g root: *χ* ^2^ = 53.33, *p* < 0.001; Figure 3a,e) or related range expanders (N/100 g soil: *χ* ^2^ = 55.49, *p* < 0.0001; N/g root: *χ* ^2^ = 97.99, *p* < 0.0001; Figure 3a,e). *Meloidogyne* densities in mesocosms with natives were higher than in mesocosms with related range expanders (N/100 g soil: *χ* ^2^ = 8.12, *p* < 0.01; N/g root: *χ* ^2^ = 6.77, *p* < 0.01; Figure 3a,e).

Soils from the original range contained more *Hoplolaimidae* (N/100 g soil: explained deviance = 13.12, *p*(*χ* ^2^, *df* = 1) < 0.001; N/g root: explained deviance = 10.64; *p*(*χ* ^2^, *df *= 1) < 0.01; Figure 3b,f) and *Tylenchidae* (N/100 g soil: explained deviance = 21.06, *p*(*χ* ^2^, *df* = 1) < 0.0001; N/g root: explained deviance = 18.02, *p*(*χ* ^2^, *df* =) < 0.0001; Figure 3c,g) than soils from the original range. The densities of *Hoplolaimidae* on roots differed also between plant communities (explained deviance = 22.83, *p*(*χ* ^2^, *df* = 2) < 0.0001; Figure 3f): unrelated range expanders had more *Hoplolaimidae* per g root than natives (*χ* ^2^ = 10.83; *p* < 0.001) and related range expanders (*χ* ^2^ = 18.67, *p* < 0.0001). *Tylenchidae* densities in soil were also affected by plant community type (explained deviance = 8.25, *p*(*χ* ^2^, *df* = 2) < 0.05; Figure 3c): both natives (*χ* ^2^ = 7.02, *p* < 0.01) and related range expanders (*χ* ^2^ = 7.92, *p* < 0.01) had higher *Tylenchidae* densities than unrelated range expanders. Neither plant community nor soil origin significantly affected numbers of *Paratylenchus* (Figure 3d,h).

## 4 DISCUSSION

Climate warming‐induced range‐expanding plant species can experience weaker negative impact in soil from the new than from the original range (De Frenne et al., 2014; Dostálek et al., 2015; van Grunsven et al., 2010; Van Nuland et al., 2017). This may be caused by the loss of belowground natural enemies, such as root‐feeding nematodes and soil‐borne pathogens, as a result of plants having higher dispersal capacities than soil biota (Berg et al., 2010; Morriën, Engelkes, Macel, Meisner, & Van der Putten, 2010). However, biogeographic studies on soil‐borne enemies along expansion gradients are scarce (Van Nuland et al., 2017), and to our knowledge, such studies are nonexistent along intracontinental latitudinal gradients. Our study shows that, differently as hypothesized, for none of the plant communities, there were evident differences in root‐feeding nematode community composition between original and new range soils, suggesting that range‐expanding plant species do not experience strong shifts in root‐feeding nematode communities as a consequence of latitudinal range expansion. Between new and original range soils, we did observe differences in the community composition based on nematode feeding types, but only for related range expanders. Therefore, our hypothesis of stronger nematode community shifts between the original and new range for unrelated range expanders than for range expanders with native relatives was not confirmed.

Plant community effects on root‐feeding nematode community composition were not the same between the ranges. Most notably, in the new range, the root‐feeding nematode community composition of unrelated range expanders was more distinct from the communities of natives and related range expanders in the original range (Figure 1b), suggesting distinct nematode responses to these phylogenetically distant plant species in the new range. Moreover, root‐feeding nematode communities of natives and related range expanders were more comparable in the new range than in the original range, suggesting nematode spillover effects from natives to related range expanders. In spite of these interactive effects between plant community and soil origin on the root‐feeding nematode community composition, we did not find such significant interaction effects on densities of root‐feeding nematodes or on root‐feeding nematode groups (Figures 2 and 3, respectively). This may indicate relatively subtle shifts in multiple root‐feeding nematode groups that only could be detected when the full root‐feeding nematode community was analyzed. Densities of Hoplolaimidae and Tylenchidae were higher in soils from the original than from the new range, but these effects did not depend on plant community (Figure 3) and therefore do not underlie the observed interactive effect. Also, the interactive effect of plant community and soil origin on the nematode community composition based on nematode feeding types could not be explained by differences in the densities of the different nematode‐feeding groups. Possibly densities of predatory nematodes play a role in the statistical separation between the original and new range for the plant community of related range expanders, but total predatory nematode densities were too low to reliably model in a univariate analysis.

The root‐feeding nematode community of unrelated range expanders differed from those of native and related range expanders. These differences in nematode community composition may be explained by plant phylogeny, as the unrelated range expanders belong to different genera than the natives and related range expanders and therefore have different traits (Gilbert & Webb, 2007). However, as the community of unrelated range expanders was largely dominated by annuals, whereas the other two communities include mostly perennials (Koorem et al., 2017), it is also possible that nematode responses were the result of differences in plant life history strategies. Annual plant species are often early successional colonizers known to develop strongly negative plant–soil feedbacks (Kardol, Bezemer, & van der Putten, 2006), which corresponds with the strong accumulation of root‐feeding nematodes found in the plant community of unrelated range expanders. While the plant species in the community of unrelated range expanders had the smallest root systems (Supporting Information Appendix S2; Koorem et al., 2017), they accumulated the highest numbers of root‐feeding nematodes, suggesting poor defense against nematodes. As a result, differences between plant communities were even stronger when nematode densities were expressed per gram of root (Figure 3).

While intercontinentally exotic early colonizers have been shown to accumulate fewer natural enemies in their new than in their original range (Blumenthal, Mitchell, Pyšek, & Jarošík, 2009), we found no such pattern in our study. Experimental comparisons between the group of unrelated range expanders and native plant species with an annual life history strategy are needed in order to examine whether there is any benefit for this group of range expanders over ecologically comparable native plant species in the new range. However, in order to examine the effects of ecological novelty associated with phylogenetic distinctiveness (Strauss et al., 2006) in the context of climate‐driven range expansion, future studies also need to focus on unrelated range expanders with a perennial life history. Overall, our results emphasize that plant species’ life histories need to be taken into account when analyzing effects of biotic interactions on range‐expanding and exotic plant species.

As hypothesized, root‐feeding nematodes were more clearly affected by the different plant communities than the other nematode‐feeding groups. The community of related range expanders accumulated fewer root‐feeding nematodes than their congeneric natives, which is in line with a study on range‐expanding plant species in their new range soil (Morriën et al., 2012). Our study, which considered responses of nematode communities from both the new and original range, shows that range expanders also accumulate fewer root‐feeding nematodes in soil from their original range than related species native in both areas of soil origin (Figure 2). These results suggest that related range expanders on average are better defended against root‐feeding nematodes than related native species, regardless of the origin of the nematodes. This corresponds with a previous study showing that intracontinental range expanders were better defended against an aboveground herbivore that was naïve to all of the examined plant species (Engelkes et al., 2008). However, all plants used in the study by Engelkes et al. (2008), as well as in the present study, originated from seeds that were collected from the new range (The Netherlands). We therefore cannot exclude that the strong defense against root‐feeding nematodes by these related range expanders is the result of natural selection during range expansion for genotypes that are especially well‐defended against generalist herbivores (Doorduin & Vrieling, 2011; Lin, Klinkhamer, & Vrieling, 2015). Future experiments using plant populations from both the original and the new ranges of the range expanders are needed in order to examine whether such shifts in plant defense traits may have occurred during climate‐driven range expansion (Macel et al., 2017).

The nematode abundances presented in our study are the net effects of bottom‐up and top‐down control by both the plants and the micro‐organisms present in the soils (Wilschut, Geisen, Ten Hooven, & van der Putten, 2016). While bottom‐up effects on nematode numbers are stronger than top‐down effects, potential differences between the plant communities in their ability to attract natural enemies of root‐feeding nematodes, such as bacteria, fungi, and protists (Geisen et al., 2015; Piskiewicz, Duyts, Berg, Costa, & van der Putten, 2007; Stirling, Smith, Licastro, & Eden, 1998), could add additional variation in root‐feeding nematode accumulation. Additionally, the presence of chewing herbivores on part of the replicates could have had an effect on nematode numbers (Wang, Biere, van der Putten, Bezemer, & Brinkman, 2017). Our experimental setup did not allow to test these effects. Nevertheless, aboveground herbivores were only present in the final 2 weeks of the experiment, making it less likely that they had a profound effect on nematode accumulation in the soils. Interestingly, plant effects strongly differed between root‐feeding nematode groups: While *Meloidogyne* and Hoplolaimidae densities strongly depended on the plant community, such differences were not found in *Paratylenchus* and *Tylenchidae*, indicating that the latter may be more generalistic and not strongly responsive to species‐specific plant traits, such as root chemistry (Wilschut et al., 2017). This could be due to their feeding strategy (Yeates et al., 1993): While *Meloidogyne* and Hoplolaimidae partly or completely feed inside the roots, *Paratylenchus* and Tylenchidae are ectoparasites or root‐hair feeders and therefore may be less affected by defense chemistry.

We conclude that there are no consistent shifts in nematode community composition between the original and new range of range‐expanding plant species and that range expanders do not accumulate fewer root‐feeding nematodes in the new range than in the original range. Unexpectedly, the range expanders without native congeners accumulated more root‐feeding nematodes than the natives and their congeneric‐related range expanders, but this might also be due to their annual life history strategy. The community of congeneric‐related range expanders was found to be the most suppressive to root‐feeding nematodes compared to the natives, which may have benefitted their range expansion. Subsequent studies are needed where plant populations from both ranges will be included in the analysis, in order to elucidate the impact of range‐expanding plant species on native soil communities.

## AUTHOR CONTRIBUTIONS

All authors contributed to the experimental design. K.K. was responsible for the main pot experiment. R.A.W. analyzed the nematode communities. O.K. contributed to statistical analyses. R.A.W. wrote the manuscript, with contributions of all other authors.

## DATA ACCESSIBILITY

Experimental data (nematode counts) are uploaded to Dryad (https://doi.org/10.5061/dryad.t1f10sr).
