# Supplementary material for: Nematode community responses to range‐expanding and native plant communities in original and new range soils
Source: Ecol Evol. 2018 Oct 2;8(20):10288–97. doi: 10.1002/ece3.4505 (PMC6206179; doi:10.1002/ece3.4505)
Supplement: Supplementary file 1 [file ECE3-8-10288-s001.pdf]

Appendix S1: overview of soil collections for original and new range soil. Coordinates for the collection sites are given for three sub-locations per collection area in each of the three countries where soil was collected. Non-sterilized soil from each sub-location was used in one of the 9 soil mixtures of either the original range (O) or the new range (N). Note that we accounted for potential abiotic between the new range and original range soil mixes by the addition of sterilized soil from the complementing range (see methods).

Range	Location ID	Sub-location	Coordinates	Mix
	Austria (AU)			
Original	1	AU 1.1	N 48.26557 E 13.24619	O1
		AU 1.2	N 48.30533 E 13.30192	O2
		AU 1.3	N 48.30513 E 13.30614	O3
	2	AU 2.1	N 48.32249 E 14.33575	O4
		AU 2.2	N 48.31162 E 14.33176	O5
		AU 2.3	N 48.32202 E 14.31814	O6
	3	AU 3.1	N 48.31063 E 14.33555	O7
		AU 3.2	N 48.31239 E 14.33491	O8
		AU 3.3	N 48.30348 E 14.33887	O9
	Slovenia (SL)			
Original	1	SL 1.1	N 46.37294 E 14.16777	O6
		SL 1.2	N 46.37294 E 14.16777	O7
		SL 1.3	N 46.37294 E 14.16777	O3
	2	SL 2.1	N 45.92891 E 15.50848	O1
		SL 2.2	N 45.92891 E 15.50848	O5
		SL 2.3	N 45.93038 E 15.49567	O2
	3	SL 3.1	N 46.13559 E 14.60972	O9
		SL 3.2	N 46.16527 E 14,75565	O8
		SL 3.3	N 45.96904 E 14.54572	O4
	The Netherlands (NL)			
New	1	NL 1.1	N 51.87657 E 6.00357	N1, N7
		NL 1.2	N 51.87937 E 6.00413	N2, N8
		NL 1.3	N 51.86766 E 5.99216	N3, N9
	2	NL 2.1	N 51.85399 E 5.88374	N1, N4
		NL 2.2	N 51.85884 E 5.88557	N2, N5
		NL 2.3	N 51.86067 E 5.89020	N3, N6
	3	NL 3.1	N 51.89423 E 5.63424	N4, N7
		NL 3.2	N 51.89265 E 5.64489	N5, N8
		NL 3.3	N 51.89569 E 5.64446	N6, N9
