# Supplementary material for: Nematode community responses to range‐expanding and native plant communities in original and new range soils
Source: Ecol Evol. 2018 Oct 2;8(20):10288–97. doi: 10.1002/ece3.4505 (PMC6206179; doi:10.1002/ece3.4505)
Supplement: Supplementary file 2 [file ECE3-8-10288-s002.pdf]

Appendix S2: Shoot and root biomass (g) of the plant communities 'natives' (NAT), 'related range-expanders' (RRE) and 'unrelated range-expanders' (URE) in original and new range soils.

	**Shoot biomass (g)**		**Root biomass (g)**	
**Community (N=9)**	Mean	±SE	Mean	±SE
NAT Original	10.93	0.44	16.64	0.76
NAT New	11.81	0.61	17.04	0.94
RRE Original	12.44	0.67	14.86	0.71
RRE New	13.33	0.64	16.22	0.47
URE Original	20.28	0.74	6.19	0.28
URE New	21.53	0.99	6.63	0.56
